# A Case of Thyroid Dermopathy

**DOI:** 10.7759/cureus.113624

**Published:** 2026-07-29

**Authors:** Andrew J Day, G. Peter Sarantopoulos

**Affiliations:** 1 Medicine, University of California Los Angeles David Geffen School of Medicine, Los Angeles, USA; 2 Pathology, University of California Los Angeles David Geffen School of Medicine, Los Angeles, USA

**Keywords:** graves disease, pretibial myxedema, thyroid dermopathy, thyroid disorder, thyroid eye disease (ted)

## Abstract

Thyroid dermopathy is a relatively uncommon extrathyroidal manifestation of autoimmune thyroid disease in which there is localized thickening of the skin due to accumulation of glycosaminoglycans. Thyroid dermopathy is classified as a cutaneous mucinosis and is thought to occur in less than 5% of all patients with Graves’ disease, most commonly in the setting of severe thyroid eye disease. We present a case of a forty-year-old woman with long-standing Graves’ disease and thyroid eye disease who developed progressive thyroid dermopathy over years. Despite partial response to intralesional glucocorticoid and hyaluronidase, definitive symptom control was not obtained until immunomodulatory therapy was started for other concomitant autoimmune conditions. Here we present a case of significant thyroid dermopathy that responded well to systemic immunomodulatory therapy that was prescribed for concomitant autoimmune conditions. This case underscores the diagnostic challenges and limited treatment options for thyroid dermopathy and discusses the potential role for systemic therapy in more advanced disease.

## Introduction

Graves’ disease is an autoimmune condition that primarily affects the thyroid gland and is the most common etiology of hyperthyroidism. Some, but not all, patients with Graves’ disease will develop extrathyroidal manifestations including thyroid eye disease, thyroid dermopathy, and/or thyroid acropachy [1,2]. Thyroid dermopathy is an uncommon extrathyroidal manifestation of Graves’ disease that is characterized by localized thickening of the skin. This thickening of the skin often impacts the pretibial region and thus is often referred to as pretibial myxedema. While thyroid dermopathy likely affects less than 5% of patients with Graves' disease, rates are considerably higher among patients with clinically significant thyroid eye disease [3]. In fact, the pathogenesis of thyroid dermopathy is thought to parallel that of thyroid eye disease with thyrotropin receptor antibody-mediated activation of TSH-receptor-expressing fibroblasts [4].

While not always associated with significant morbidity, thyroid dermopathy can cause pain, functional impairment, and disfigurement. Despite this, thyroid dermopathy often remains underrecognized and difficult to manage. The diagnosis is largely clinical. Existing therapy options are largely palliative. There are no current FDA-approved therapies for this condition, and there are no randomized-controlled trials to help guide management decisions. Here we present a case of severe thyroid dermopathy that resulted in significant pain and functional impairment and did not respond initially to intralesional therapy, though it ultimately did respond to a multi-drug immunomodulatory regimen that was prescribed for the management of concomitant autoimmune conditions. This case illustrates the diagnostic and therapeutic challenges presented by thyroid dermopathy.

## Case presentation

**Figure 1:**
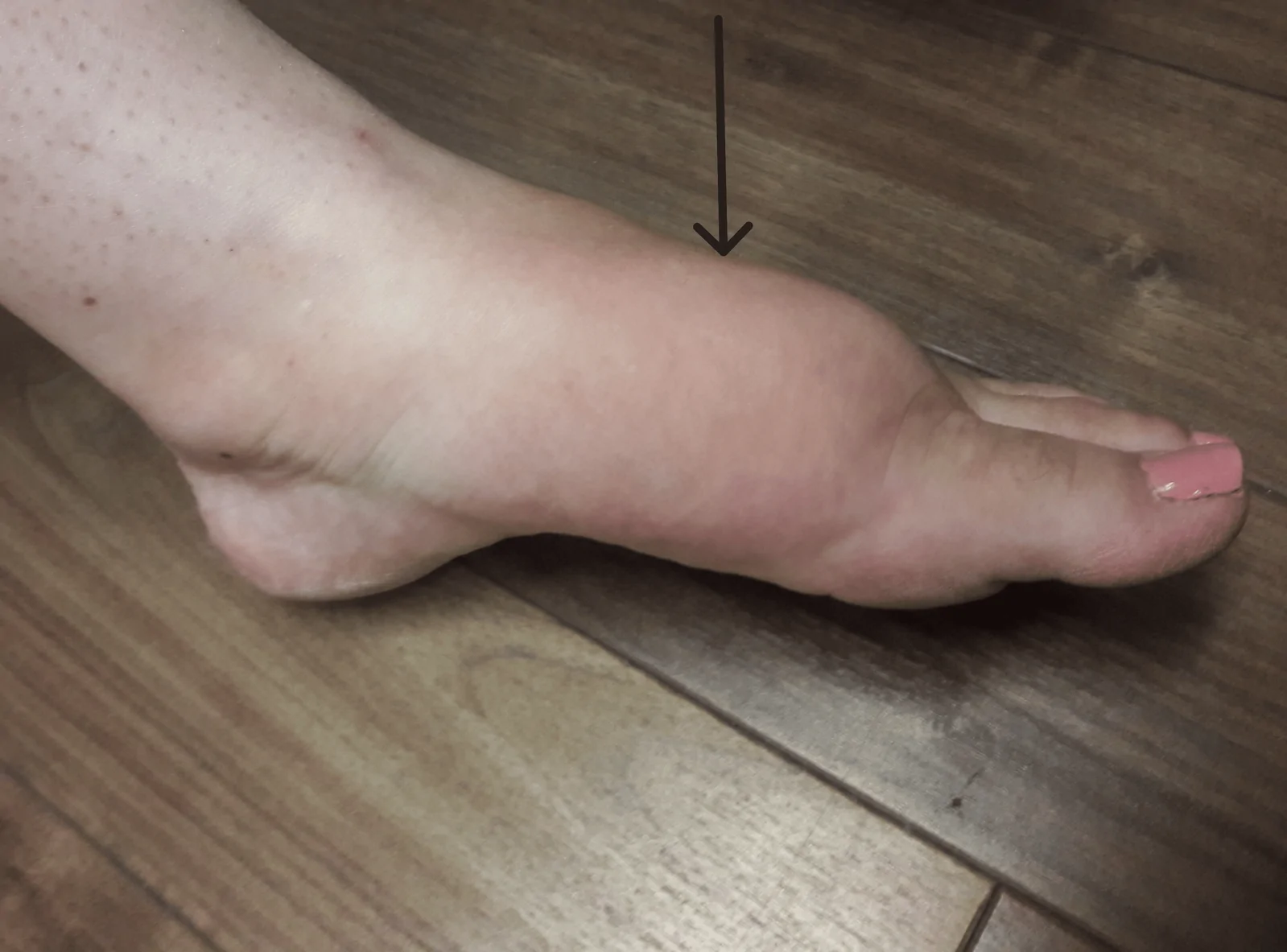
Clinical Photograph The arrow indicates the indurated plaque and edema involving the majority of the dorsum of the foot.

A 40-year-old woman presented to the endocrine clinic to establish care for management of her thyroid condition. Eleven years before this visit, she was diagnosed with hyperthyroidism from Graves’ disease. She had never used tobacco products. Her condition was complicated by thyroid eye disease. The hyperthyroidism was initially managed with methimazole. Four years after the initial diagnosis, she did become pregnant. The pregnancy went well; however, afterwards her thyroid eye disease worsened, and the hyperthyroidism became more difficult to manage with medication. She ultimately underwent total thyroidectomy. She also required multiple orbital decompression surgical procedures for the thyroid eye disease, which did not resolve following thyroidectomy. At the current visit, the patient mentioned bilateral foot pain and swelling that had been progressive for at least five years. She had to change shoe sizes multiple times. She had a previous MRI report that mentioned: “diffuse T2 hyperintense reticular change throughout the dorsum of the foot.” Physical examination of the foot and pretibial region revealed large, indurated plaques and non-pitting edema (Figure 1) that involved the entire dorsum of the foot. Thyroid function tests obtained during this visit were within normal ranges, though thyrotropin receptor autoantibodies remained quite elevated (Table 1). She was given a clinical diagnosis of thyroid dermopathy and referred to dermatology for confirmation of diagnosis and potential treatment.

**Table 1:** Laboratory Results

Variable	Result	Reference Range
Thyroid Stimulating Immunoglobulins	>500%	≤122%
Thyrotropin Receptor Antibody	>40.00 IU/L	≤1.75 IU/L
Thyrotropin Binding Inhibitory Immunoglobulins	92%	≤16% inhibition

**Figure 2:**
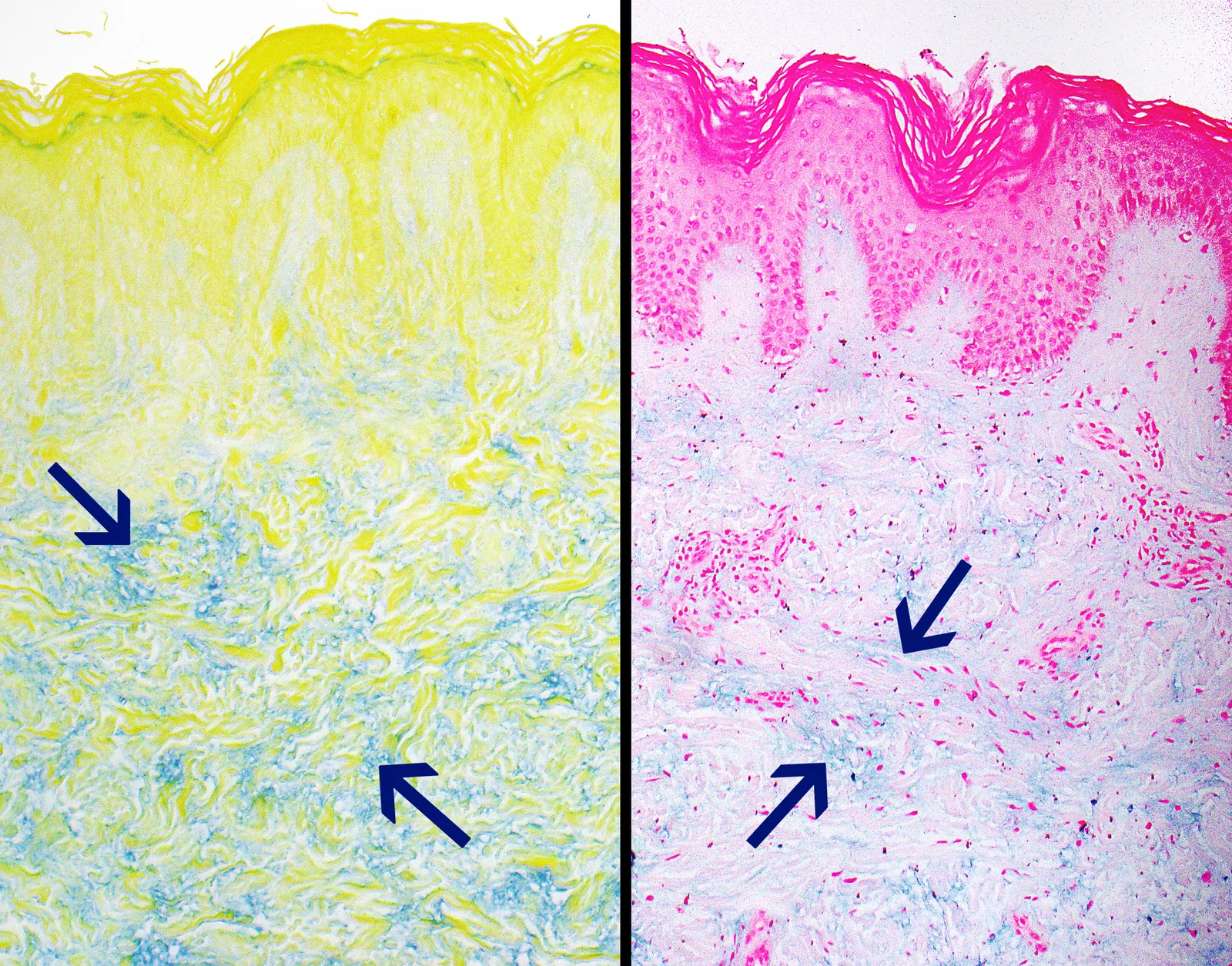
Histologic Sections with Specialized Mucin Staining Special staining highlights well-dispersed blue staining of superficial to deep interstitial mucin (left pane colloidal iron, right pane alcian blue @ 2.5 ph, 100x). Arrows refer to examples of the diffuse blue staining.

Punch biopsy of the skin overlying the left dorsal foot revealed mild perivascular lymphocyte-predominant superficial perivascular inflammation and increased interstitial mucin (Figure 2). The patient did not want to apply topical corticosteroids and elected to proceed with intralesional injection of triamcinolone acetonide and hyaluronidase. With minimal improvement in symptoms, oral pentoxifylline was prescribed, though the patient did not feel comfortable and elected to not take the medication.

## Discussion

Thyroid dermopathy is a relatively uncommon, extrathyroidal manifestation of autoimmune thyroid disease in which there is localized thickening of the skin. Pretibial (or localized) myxedema is a term that is often used interchangeably. Myxedema is derived from the Greek mýxa (mucus) and oidēma (swelling).

Thyroid dermopathy is classified as a cutaneous mucinosis [5]. Histologic features of thyroid dermopathy are similar to those of thyroid eye disease and include excess accumulation of glycosaminoglycans in the reticular dermis as well as lymphocyte infiltration in the perivascular spaces. Mucinous material will often cause disruption of collagen bundles in the connective tissue.

The exact mechanisms underlying the pathogenesis of thyroid dermopathy are not fully understood, though the pathogenesis is thought to be closely related to and similar to that of thyroid eye disease [4]. An interaction of immunologic, cellular, and mechanical processes seems to result in the clinical manifestations of thyroid dermopathy. Classically, it has been felt that autoantibodies in the serum of patients with Graves’ disease interact with a common antigen in the thyroid gland and connective tissue, presumably thyrotropin receptors. Thyrotropin receptor mRNA has, in fact, been detected in fibroblasts cultured from thyroid dermopathy lesions. This interaction then initiates an immune cascade that ultimately leads to the activation of fibroblasts, resulting in excessive production of glycosaminoglycans. Glycosaminoglycan accumulation can expand the dermal tissue, leading to fluid accumulation. It is thought that this becomes clinically apparent in the setting of local factors such as dependent position (venous stasis), mechanical stress, and/or trauma [6]. Secondary lymphatic obstruction and fibrosis can result in worsening of the lesions in more advanced cases. Thyroid hormone status does not appear to correlate with cutaneous symptoms. More recently, a pathway involving the IGF-1 receptor as a mediator of this pathogenic process has been proposed [2].

It has been noted that almost all patients with thyroid dermopathy will have very high levels of circulating thyrotropin receptor autoantibodies and will have thyroid eye disease, often severe [7,8]. The onset of thyroid dermopathy usually occurs after the onset of thyroid eye disease and, on average, occurs 12 to 24 months after the diagnosis of thyrotoxicosis. These consistent clinical findings seem to suggest that both the severity of the autoimmune process and the disease duration are important for the development of the dermopathy features.

Skin lesions are often symmetric and limited to the pretibial region, though, rarely, involvement of the hands, face, abdomen, scalp, shoulder, and forearm has been observed. Often there is a history of trauma when lesions are found in these less common locations. Various clinical forms have been described, including a non-pitting edema form, a plaque form, a nodular form, and an elephantiasis form [5]. The non-pitting edema form is most common. In this form, skin lesions are often described as firm or indurated, raised, and waxy. Lesions are often flesh-colored, though may be pigmented. Hyperhidrosis is common, and prominent hair follicles can give lesions a peau d'orange appearance. The plaque form often exhibits raised plaques on a background of non-pitting edema. The nodular form has sharply circumscribed nodular or tubular lesions. The elephantiasis form is more severe and will often have nodular lesions, fibrosis of the skin, and significant lymphedema. About 20% of patients with thyroid dermopathy will also have thyroid acropachy. Thyroid dermopathy is typically only of cosmetic concern. In fact, skin lesions often go unnoticed as more attention is placed on the thyrotoxicosis and thyroid eye disease. Rarely, the skin lesions will be painful or pruritic. More advanced lesions in the hands or feet can result in functional problems.

Thyroid dermopathy is thought to occur in less than 5% of all patients with Graves’ disease and in about 13-15% of patients with significant thyroid eye disease [8]. Subclinical forms may be more common. In a large case series, 80% of patients were women, and a history of tobacco use was present in 75% of patients.

Thyroid dermopathy is typically going to be a clinical diagnosis, especially when pretibial skin lesions occur in the setting of thyroid eye disease and elevated thyrotropin receptor autoantibodies. Skin biopsy can be considered if the diagnosis is in doubt. MRI findings have been described, though imaging studies are rarely helpful or necessary.

At the current time, there are no Food and Drug Administration (FDA)-approved treatments for thyroid dermopathy, and there are no randomized controlled trials to guide treatment decisions [3,4]. It is presumed that obtaining euthyroid status, tobacco cessation, obtaining a healthy body weight, and avoiding skin trauma and venous stasis may help prevent the development of thyroid dermopathy. It is not known whether choosing a particular form of treatment for the thyrotoxicosis (i.e., antithyroid medication, radioiodine ablation, or thyroidectomy) offers benefits in terms of dermopathy outcomes. Observation may be appropriate for mild cases, though treatment can be considered if there are cosmetic concerns or discomfort. It is plausible that initiating treatment early may help prevent more advanced disease. Current treatment options are generally considered to be symptomatic or palliative rather than curative. If treatment is considered, the treatment of choice has long been topical corticosteroids under an occlusive dressing, such as plastic wrap. Intralesional therapy with corticosteroids, hyaluronidase, or octreotide has been used with variable results; these may be more helpful in the plaque or nodular forms. Surgery should generally be avoided given high rates of recurrence and the possibility of trauma contributing to aggravation of dermopathy lesions. Systemic immunomodulation is rarely needed for thyroid dermopathy alone. That being said, thyroid dermopathy is often associated with severe thyroid eye disease. As systemic immunomodulation can be considered for severe thyroid eye disease, there is some experience with therapies such as intravenous immunoglobulin, plasmapheresis, pentoxifylline, octreotide, rituximab, and tumor necrosis factor inhibitors in patients with thyroid dermopathy. This outcome data is generally of low quality. In 2020, the FDA approved teprotumumab-trbw, a monoclonal antibody that blocks activation of the IGF-1 receptor, for the treatment of thyroid eye disease. This is the first time a drug has been specifically approved for the treatment of an extrathyroidal manifestation of Graves’ disease. Since this approval, case reports have demonstrated improvement in thyroid dermopathy lesions in patients who have received teprotumumab-trbw for thyroid eye disease [9,10]. Little is known about long-term outcomes.

## Conclusions

The patient continued intralesional steroid injections for three years until she was diagnosed with myasthenia gravis. As treatment for the myasthenia gravis she did receive oral prednisone and mycophenolate. Intravenous immunoglobulin infusions were later added when it was felt that she may have stiff-person syndrome as well. She noticed significant subjective improvement in her lower extremity pain and swelling on this multi-drug regimen.

In summary, this present case illustrates important lessons regarding the extrathyroidal manifestations of Graves’ disease. Thyroid dermopathy tends to occur after the development of thyrotoxicosis and almost always occurs in the setting of severe thyroid eye disease. Thyroid dermopathy is associated with high levels of thyrotropin receptor autoantibodies and could be thought of as a marker of more severe autoimmune thyroid disease. While dermopathy-specific treatment may not always be necessary, it may be reasonable to consider early topical corticosteroid therapy under an occlusive dressing to prevent more advanced features as current treatment options are limited for more advanced disease. This current case, in additional other published case reports, possibly suggests that systemic immunomodulation may be helpful for advanced cases of thyroid dermopathy. It should be stated, however, that there is not strong high-quality data to strongly support its use. While randomized controlled trials would certainly help guide more advanced treatment choices, these may not be possible given the rarity of the condition and also the diagnostic challenges and heterogeneity of the condition. 
